# Colonization of Enteroaggregative *Escherichia coli* and Shiga toxin-producing *Escherichia coli* in chickens and humans in southern Vietnam

**DOI:** 10.1186/s12866-016-0827-z

**Published:** 2016-09-09

**Authors:** Nguyen Vinh Trung, Hoang Ngoc Nhung, Juan J. Carrique-Mas, Ho Huynh Mai, Ha Thanh Tuyen, James Campbell, Nguyen Thi Nhung, Pham Van Minh, Jaap A. Wagenaar, Nguyen Thi Nhu Mai, Thai Quoc Hieu, Constance Schultsz, Ngo Thi Hoa

**Affiliations:** 1 Department of Medical Microbiology, Academic Medical Center, University of Amsterdam, Amsterdam, The Netherlands; 2 Department of Global Health-Amsterdam Institute for Global Health and Development, Amsterdam, The Netherlands; 3 Oxford University Clinical Research Unit, Centre for Tropical Medicine, Ho Chi Minh City, Vietnam; 4 Centre for Tropical Medicine, Nuffield Department of Medicine, University of Oxford, Oxford, UK; 5 Sub-Department of Animal Health, My Tho, Tien Giang Vietnam; 6 Department of Infectious Diseases and Immunology, Faculty of Veterinary Medicine, Utrecht University, Utrecht, The Netherlands; 7 Central Veterinary Institute of Wageningen UR, Lelystad, The Netherlands; 8 Preventive Medicine Center, My Tho, Tien Giang Vietnam

**Keywords:** EAEC, STEC, *E. coli*, Chicken, Humans, Vietnam

## Abstract

**Background:**

Enteroaggregative (EAEC) and Shiga-toxin producing *Escherichia coli* (STEC) are a major cause of diarrhea worldwide. *E. coli* carrying both virulence factors characteristic for EAEC and STEC and producing extended-spectrum beta-lactamase caused severe and protracted disease during an outbreak of *E. coli* O104:H4 in Europe in 2011. We assessed the opportunities for *E. coli* carrying the *aggR* and *stx* genes to emerge in ‘backyard’ farms in south-east Asia.

**Results:**

Faecal samples collected from 204 chicken farms; 204 farmers and 306 age- and gender-matched individuals not exposed to poultry farming were plated on MacConkey agar plates with and without antimicrobials being supplemented. Sweep samples obtained from MacConkey agar plates without supplemented antimicrobials were screened by multiplex PCR for the detection of the *stx1*, *stx2* and *aggR* genes. One chicken farm sample each (0.5 %) contained the *stx1* and the *aggR* gene. Eleven (2.4 %) human faecal samples contained the *stx1* gene, 2 samples (0.4 %) contained *stx2* gene, and 31 (6.8 %) contained the *aggR* gene. From 46 PCR-positive samples, 205 *E. coli* isolates were tested for the presence of *stx1*, *stx2*, *aggR*, *wzx*_O104_ and *fliC*_H4_ genes. None of the isolates simultaneously contained the four genetic markers associated with *E. coli* O104:H4 epidemic strain (*aggR*, *stx2*, *wzx*_*O104*_ and *fliC*_*H4*_). Of 34 EAEC, 64.7 % were resistant to 3^rd^-generation cephalosporins.

**Conclusion:**

These results indicate that in southern Vietnam, the human population is a more likely reservoir of *aggR* and *stx* gene carrying *E. coli* than the chicken population. However, conditions for transmission of isolates and/or genes between human and animal reservoirs resulting in the emergence of highly virulent *E. coli* strains are still favorable, given the nature of‘backyard’ farms in Vietnam.

**Electronic supplementary material:**

The online version of this article (doi:10.1186/s12866-016-0827-z) contains supplementary material, which is available to authorized users.

## Background

*Escherichia coli* is one of the most widely distributed bacteria in the environment, and therefore humans and animals are exposed to saprophytic *E. coli* strains throughout their lives. Some *E. coli* strains may become capable of causing disease in humans and some animal species by expression of one or multiple virulence factors, such as adhesins and toxins [1]. These virulence factors are encoded by genes which are typically located on mobile genetic elements such as plasmids or phages. Horizontal gene transfer between *E. coli* strains carrying different virulence determinants may result in novel virulence gene combinations leading to highly virulent phenotypes.

An example of the emergence of highly virulent pathogenic *E. coli* strains is the Enterohaemorrhagic *E. coli* (EHEC) O104:H4, responsible for the large and devastating outbreak of hemorrhagic colitis in Europe in 2011 [2]. This outbreak strain was found to carry an unusual combination of the pathogenic features of enteroaggregative *E. coli* (EAEC) and Shiga toxin (Stx)-producing *E. coli* (STEC). Among the 6 pathotypes of *E. coli* capable of causing enteric diseases, EAEC has emerged as a cause of acute and persistent diarrhea in children, as well as adults, in both developed and developing countries [3, 4]. EAEC has rarely been isolated from animal sources such as dogs and cats [5], but whether animals are reservoirs of EAEC or are accidental hosts of EAEC due to close contact with humans [6] remains to be determined. In contrast, it has been shown that cattle are the major reservoir species of STEC. However, other livestock species, including sheep, goats, horses, pigs, and water buffalo, are also capable of harboring these organisms [7]. In fact, STEC has been found in contaminated food, water [8] and the farm environment [9–11]. Given these differences between reservoirs, it is striking that Stx-producing EAEC has emerged to cause outbreaks in humans [12]. Therefore, further investigation of the distribution of genes encoding for virulence factors *aggR*, *stx1* and *stx2* in environmental, animal and human reservoirs is required to better understand the potential for the emergence of *E. coli* carrying this unusual combination of virulence genes.

Although the prevalence of STEC in chicken is considered minimal, a previous study has shown that chickens are readily and persistently infected by STEC [13]. Small-scale and backyard chicken farming are very common in southeast Asia, including the Mekong Delta in southern Vietnam [14, 15]. In such farms, there is typically a great degree of overlap between the farming and household environments, providing ample opportunity for horizontal gene transfer between human and poultry *E. coli*. We aimed to assess the potential of such an occurance, by investigating the prevalence of the highly conserved *aggR* and the *stx1, stx2* genes in samples collected from chicken farms, farmers and in matched asymptomatic humans not exposed to chickens, as well as in *E. coli* isolates from these samples. In addition, with the hypothesis that *E. coli* O104:H4 epidemic strains may be present in Vietnam, we also screened for the presence of the combination of *aggR*, *stx2, wzx*_O104_ and *fliC*_H4_ genes in *E. coli* isolates.

## Methods

### Sample collection

This study was carried out as a part of a larger study on antimicrobial drug usage and antimicrobial resistant *E. coli* colonization in backyard chicken farms and humans. We collected faecal swabs from 204 randomly selected chicken farms; 204 healthy farmers in those farms, and 306 age- and sex-matched individuals in Tien Giang province, Vietnam. The matched individuals not involved in poultry farming were randomly selected from the same district as the farmers (rural individual, *N* = 204,), as well as from the provincial capital (urban individual, *N* = 102), using the population census of Tien Giang [16]. Farms and household visits were evenly distributed over a 13-month-period from March 2012 to April 2013 in order to avoid seasonal effects. Faecal samples from chickens were collected using boot-swabs or hand-held gauze swabs, as described previously [17]. The chicken sample collection was conducted by a trained sampling team from the Tien Giang Sub-Department of Animal Health. Rectal swab samples were obtained from all human participants by trained staff from Tien Giang Preventive Medicine Center, using Fecalswab (Copan, Italy). All samples were stored and transported at 4oC to the laboratory at the Oxford University Clinical Research Unit in Ho Chi Minh City and cultured within 24 h after sample collection.

### *E. coli* isolation and antimicrobial susceptibility testing

Buffered Peptone Water (225 mL) was added to each chicken faecal sample in a separate container and was manually shaken. A volume of 1 mL from each container was diluted 1:1000 in saline solution. Human rectal swabs were vortexed to release and suspend the sample in the liquid transport medium and then 100 μL was diluted 1:100 in saline solution. A volume of 50 μL of each saline diluted sample was plated onto MacConkey agar (Oxoid, UK) with or without antimicrobials supplemented. This procedure yielded on average 100 single colonies on each plate, which allowed for unbiased detection of *E. coli*-like colonies of multiple morphologies in all samples. A random selection of 5 (from MacConkey agar plate without antimicrobial supplemented) and 2 (from each of MacConkey agar plates supplemented with either nalidixic acid [16 mg/L], ceftazidime [2 mg/L], or gentamicin [8 mg/L]) presumptive *E. coli*-like colonies were sub-cultured, identified and tested for their susceptibility using disc diffusion method in accordance with the Clinical and Laboratory Standards Institute guidelines [18]. Eleven antimicrobials (all Oxoid, UK) were tested including tetracycline (30 mg), trimethoprim/sulfamethoxazole (1.25/23.75 mg), chloramphenicol (30 mg), gentamicin (10 mg), amikacin (30 mg), ciprofloxacin (5 mg), ampicillin (10 mg), amoxicillin/clavulanic acid (30 mg), ceftazidime (30 mg), ceftriaxone (30 mg) and meropenem (10 mg). Quality controls for susceptibility testing and identification were performed every week according to the CLSI guidelines. Strains with an intermediate susceptible result were considered resistant. An MDR strain was defined as a strain resistant to at least three different antimicrobial classes.

Only *E. coli* strains with a unique morphology and/or phenotypic antimicrobial susceptibility pattern isolated from each sample were saved for further analyses. In addition, a sweep from the full remaining growth on the MacConkey plate without antimicrobial supplement was collected, suspended in glycerol and stored in screw-cap tubes at - 20 °C for further analyses.

### Screening for the presence of *E. coli* virulence factors

As using sweep samples for PCR screening has been shown to be efficient and sensitive [19], multiplex PCR was first performed on the glycerol stored sweeps to screen for the presence of *aggR*, *stx1* and *stx2* genes. In brief, one loopful (1 μl) of the −20 °C stored sweep was collected and cultured on the MacConkey agar, then incubated for 16 h at 37 °C. A sweep of the bacterial growth was then collected and suspended in 1 ml of water. Suspensions were heated at 95 °C for 3 min and immediately placed on ice then centrifuged at 9000 rpm/ 3 min to collect DNA in the supernatant. Primers used for the multiplex PCR are listed in Table 1*.* Primers were designed by using Nucleotide Blast, Align X and OligoAnalyzer 3.1 with the published sequences on NCBI database (Genbank accession numbers are M19473, X07865, Z18751 for *stx1*, *stx2* and *aggR*, respectively). The detection limit of the assay was determined by cloning the target sequences into a pCR2.1 plasmid (TA Cloning kit, Invitrogen, USA). The PCR had a detection limit of 10 copies per ml for each of the target gene as determined by serial dilution from 10 ng/μl to 10^-6^ ng/μl. Each reaction mixture contained 2 mM MgCl_2_, 0.1 mM deoxyribonucleotides, 0.2 uM each of the oligonucleotides and 0.5 U of Taq polymerase (Bioline, UK), to which 50 – 100 ng of the DNA suspension was added. The mixtures were processed in a GeneAmp PCR system 9700 (Applied Biosystems, USA). The PCR program was 96 °C for 4 min, 30 cycles of 94 °C for 20 s, annealing at 55 °C for 20 s, and 72 °C for 10 s. The final extension step was 72 °C for 7 min. DNA extracted from *E. coli* E3787 (*stx1*), *E. coli* E32511 (*stx2*), *E. coli* E69187 (*aggR*) was used as a positive control in PCR reactions. A negative control, containing water instead of template DNA was included in each run of PCR reactions.

**Table 1:** List of primers used in this study

Target genes	Primer name	Primer sequences (5′-3′)	Fragment size	Reference
*aggR*	aggR_F	AAGCAGCGATACATTAAGACG	424	This study
	aggR_R	TGCTTTGCTCATTCTTGATTGC		
*stx1*	stx1_F	TGATGATTGATAGTGGCACAGG	299	This study
	stx1_R	AGAAGTAGTCAACGAATGGCG		
*stx2*	stx2_F	ACATCGGTGTCTGTTATTAACC	666	This study
	stx2_R	TTGACTCTCTTCATTCACGGC		
*wzx*_O104_	wzx_O104__F	GGTTTTATTGTCGCGCAAAG	337	[20]
	wzx_O104__R	TATGCTCTTTTTCCCCATCG		
*fliC*_H4_	fliC_H4__F	ACGGCTGCTGATGGTACAG	244	[20]
	fliC_H4__R	CGGCATCCAGTGCTTTTAAC		

From any sweep sample which produced positive PCR result for any of the target genes in multiplex PCR, DNA extracted from all stored *E. coli* isolates obtained after culture of the corresponding faecal sample on MacConkey agar plates with and without antimicrobials supplemented, was subsequently tested for the presence of 3 genes (*aggR*, *stx1* and *stx2*) using multiplex PCR. The presence of 2 genes (*wzx*_O104_ and *fli*C_H4_) was also investigated using monoplex PCR [20].

## Results

Sweep samples were available for 188 of 204 chicken farms, 186 of 204 farmers, 182 of 204 rural individuals and 90 of 102 urban individuals. The remaining sweep samples were either missing (16 chicken samples and 45 human samples) or were not available because the primary faecal culture on MacConkey agar did not show any growth (7 human samples). The *aggR* gene was detected in 31 (6.8 %) human samples but in only 1 (0.5 %) of the chicken farm samples (*p* < 0.001). Gene *stx1* was detected in 11 human samples (2.4 %) and in one chicken farm sample (0.5 %) whereas gene *stx2* was detected in one farmer (0.5 %) and in one rural individual (0.5 %). In 46 samples, at least one of three tested genes (*aggR, stx1* and *stx2*) was detected in the multiplex PCR. None of the samples was positive with multiple genes (Table 2).

**Table 2:** Prevalence of *aggR*, *stx1* and *stx2* genes in sweep samples and prevalence of *aggR*, *stx1*, *stx2, wzx*_O104_ *and fliC*_H4_ *genes* in *E. coli* isolated from chicken faecal samples and human rectal swabs

Subject	No. of positive samples (%)			No. of positive *E. coli* / No. of *E. coli* investigated (%)				
	*aggR*	*stx1*	*stx2*	*aggR*	*stx1*	*stx2*	*wzx*_O104_	*fliC*_H4_
Chicken farm (*N* = 188)	1 (0.5)	1 (0.5)	0	0/4	0/5	ND	0/9	1/9 (11.1)
Farmer (*N* = 186)	12 (6.5)	7 (3.8)	1 (0.5)	16^a^/55 (29.1)	1/29 (3.4)	0/4	1/88 (1.1)	12/88 (13.6)
Rural individual (*N* = 182)	13 (7.1)	3 (1.6)	1 (0.5)	16^b^/58 (27.6)	0/13	0/4	0/75	5/75 (6.7)
Urban individual (*N* = 90)	6 (6.7)	1 (1.1)	0	2^c^/27 (7.4)	0/6	ND	0/33	1/33 (3.0)

*ND* not done

^a^ *aggR*-positive *E. coli* were isolated from samples of 7 farmers

^b^ *aggR*-positive *E. coli* were isolated from samples of 7 rural individuals

^c^ *aggR*-positive *E. coli* were isolated from sample of 1 urban individual

DNA samples of 205 *E. coli* isolated from those 46 MacConkey sweep samples were tested for the presence of *aggR, stx1*, *stx2* and then further screened for the presence of *wzx*_O104_ and *fliC*_H4_ gene. The *aggR* gene was detected in 29.1, 27.6 and 7.4 % of *E. coli* strains isolated from *aggR* positive samples in farmers, rural individuals and urban individuals, respectively (Table 2). EAEC could be isolated from faecal samples of 3.8 % (7/186) of farmers, 3.8 % (7/182) rural individuals and 1.1 % (1/90) urban individuals. The EAEC isolates exhibited resistance against ampicillin (100 %), co-trimoxazole (85.3 %), tetracycline (70.6 %), gentamicin (70.6 %), ceftriaxone (64.7 %), ceftazidime (50.0 %), chloramphenicol (38.2 %), ciprofloxacin (26.5 %) and amikacin (2.9 %). 88.2 % and 50.0 % of EAEC isolates were multi-drug resistant and extended-spectrum beta-lactamase positive, respectively (Table 3).

**Table 3:** Antimicrobial susceptibility of EAEC isolates from asymptomatic humans in southern Vietnam

Antimicrobial	No. of antimicrobial resistant EAEC (%)			
	Chicken farmer	Rural individual	Urban individual	Total
	(*N* = 16)	(*N* = 16)	(*N* = 2)	(*N* = 34)
Tetracycline	8 (50.0)	14 (87.5)	2 (100)	24 (70.6)
Trimethoprim/sulfamethoxazole	13 (81.2)	14 (87.5)	2 (100)	29 (85.3)
Chloramphenicol	11 (68.8)	2 (12.5)	0	13 (38.2)
Gentamicin	13 (81.2)	11 (68.8)	0	24 (70.6)
Amikacin	0	1 (6.2)	0	1 (2.9)
Ciprofloxacin	0	9 (56.2)	0	9 (26.5)
Ampicillin	16 (100)	16 (100)	2 (100)	34 (100)
Amoxicillin/clavulanic acid	8 (50.0)	7 (43.8)	2 (100)	17 (50.0)
Ceftazidime	6 (37.5)	11 (68.8)	0	17 (50.0)
Ceftriaxone	8 (50.0)	14 (87.5)	0	22 (64.7)
Third-generation cephalosporins	8 (50.0)	14 (87.5)	0	22 (64.7)
ESBL-producing	6 (37.5)	11 (68.8)	0	17 (50.0)
Meropenem	0	0	0	0
MDR	13 (81.2)	15 (93.8)	2 (100)	30 (88.2)

*MDR* multi-drug resistant

From the 12 *stx1* positive sweep samples, only one *E. coli* (3.4 %) from a farmer, among 53 *E. coli* isolates investigated, was *stx1* positive (Table 2). This *E. coli* isolate was also multi-drug resistant against chloramphenicol, sulfamethoxazole-trimethoprim, ampicillin, tetracycline and ciprofloxacin). Detailed information on antimicrobial resistance patterns of EAEC and STEC isolated from humans in southern Vietnam is shown in Additional file 1: Table S1. Eight *E. coli* isolates from the two *stx2* PCR positive samples were tested and none showed a positive result for *stx2* gene. As a result, STEC was isolated in 0.5 % (1/186) of farmers or 0.2 % (1/458) of studied humans.

Among 196 *E. coli* isolates from 44 human individuals that were positive for any of the genes screened for using the multiplex PCR, *fliC*_H4_ gene was detected in 13.6, 6.7 and 3.0 % of *E. coli* isolates from farmers, rural individuals and urban individuals, respectively (Table 2). One out of 9 isolates from two farm samples (11.1 %) was positive with *fliC*_H4_ gene. Gene *wzx*_*O104*_ was observed in one isolate from a farmer (1.1 %). The only gene combination that we observed in this study was *aggR* and *fliC*_H4,_ which was detected in two isolates from one rural individual. None of the isolates analyzed in our study simultaneously contained the four genetic markers associated with the O104:H4 epidemic strain (*aggR, stx2*, *wzx*_O104_ and *fliC*_H4_).

## Discussion

The differences in detection of the *aggR* gene in chicken faecal samples (1; 0.5 %) and samples from asymptomatic humans (31; 6.8 %) suggest that humans are likely the main reservoir of EAEC in the setting studied. The isolation rate of EAEC from humans in our study (3.3 %, 15/458) was similar to previous studies including asymptomatic adults and children performed in northern Vietnam [21, 22]. To our knowledge, this is the first study describing the presence of the *aggR* gene in chicken faecal samples in Vietnam. We, however, speculate that this gene may have a human origin since a relatively high proportion of households in the rural areas of the Mekong Delta do not have latrines that meet established hygiene standards with respect to their construction, operation and maintenance [23].

Gene *stx1* was also detected more frequently in samples from humans compared with those from chickens (2.4 % versus 0.5 %) and the more toxic gene–*stx2*–was only detected in two samples from humans (0.4 %). The nondetection of STEC in chicken farms is in agreement with previous studies in chickens in the United States and United Kingdom in which the prevalence of STEC colonization ranged between 0 and 1.5 % [24]. Although we did not find the presence of STEC in chicken samples in this study, STEC is still an important cause of diarrhea in farm animals in Vietnam as has been shown in previous studies [25, 26]. The isolation rate of STEC (0.2 %, 1/458) and EAEC (3.3 %, 15/458) in asymptomatic humans in Vietnam is also similar to the reported prevalence in both developed and developing countries [27–29]. Our results indicate a generally high level of antimicrobial resistance among EAEC isolates from Vietnam compared with results from other countries [30] and overuse of antimicrobials in the community could be one of the possible explanations [31].

A total of 150 (73.2 %) out of 205 *E. coli* isolates, which were isolated from positive sweep samples, were negative in the colony PCR for detection of the five genes of interest. The lower isolation rate of EAEC and STEC in comparison to the *stx* and *aggR* gene detection rate on sweep samples may be due to the presence of the *stx* or *aggR* genes in *E. coli* strains other than those selected or in bacterial species other than *E. coli* but capable of growing on MacConkey agar [32]. In addition, the bacterial sweep was used as it was previously shown to better detect *E. coli* in stool samples than testing up to 5 *E. coli* isolates because of its enrichment [19]. Hence differences in sensitivity can also explain lower detection rates when testing single colonies and the absence of *stx*-positive *E. coli* amongst the limited number of isolates tested, does not completely rule out the presence of STEC in the sample.

The full combination of the four typical markers of the 2011 German *E. coli* O104:H4 outbreak strain *stx2*, *wzx*_O104_, *fliC*_H4_ and *aggR* was not detected in any *E. coli* isolated from human faecal and chicken farm samples in the current study, despite the great degree of overlap between the farming and living environment in the study setting in Vietnam. We found one combination of EAEC and STEC O104:H4 associated *aggR* and *fliC*_H4_ genes in two *E. coli* isolates from one rural individual not exposed to poultry farming.

## Conclusions

Our results indicate that in southern Vietnam, the human population is a more likely reservoir of *aggR* and *stx* gene carrying *E. coli* than the chicken population. However, it is important to note that all four typical markers of the outbreak strain (*stx2*, *wzx*_O104_, *fliC*_H4_ and *aggR*) were detected, albeit at low numbers, in samples (*stx2* and *aggR*) and *E. coli* isolates (*wzx*_O104_, *fliC*_H4_) in different host populations. Given the nature of ‘backyard’ farms in Vietnam, conditions for transmission of isolates and/or genes between human and animal reservoirs are still favorable. Therefore, strict personal hygiene practices as well as applying biosecurity in animal farming are essential to avoid the emergence of highly virulent strains.

## Additional file

Additional file 1: Table S1. Patterns of antimicrobial resistance in EAEC and STEC isolated from humans in southern Vietnam. (XLSX 10 kb)

## Abbreviations

EAEC: Enteroaggregative *Escherichia coli*
EHEC: Enterohaemorrhagic *Escherichia coli*
PMC: Preventive Medicine Centre of Tien Giang
SDAH: Sub-Department of Animal Health of Tien Giang
STEC: Shiga toxin (Stx)-producing *Escherichia coli*
